# BayesianSpikeFusion: accelerating spiking neural network inference via Bayesian fusion of early prediction

**DOI:** 10.3389/fnins.2024.1420119

**Published:** 2024-08-05

**Authors:** Takehiro Habara, Takashi Sato, Hiromitsu Awano

**Affiliations:** Department of Communications and Computer Engineering, Graduate School of Informatics, Kyoto University, Kyoto, Japan

**Keywords:** spiking neural network, Bayesian inference, neuromorphic computing, image classification, spiking network conversion

## Abstract

Spiking neural networks (SNNs) have garnered significant attention due to their notable energy efficiency. However, conventional SNNs rely on spike firing frequency to encode information, necessitating a fixed sampling time and leaving room for further optimization. This study presents a novel approach to reduce sampling time and conserve energy by extracting early prediction results from the intermediate layer of the network and integrating them with the final layer's predictions in a Bayesian fashion. Experimental evaluations conducted on image classification tasks using MNIST, CIFAR-10, and CIFAR-100 datasets demonstrate the efficacy of our proposed method when applied to VGGNets and ResNets models. Results indicate a substantial energy reduction of 38.8% in VGGNets and 48.0% in ResNets, illustrating the potential for achieving significant efficiency gains in spiking neural networks. These findings contribute to the ongoing research in enhancing the performance of SNNs, facilitating their deployment in resource-constrained environments. Our code is available on GitHub: https://github.com/hanebarla/BayesianSpikeFusion.

## 1 Introduction

In recent years, there has been extensive research on neural networks that aim to mimic the human brain. Notably, in fields like image classification and object recognition, state-of-the-art neural networks such as YOLO (Redmon et al., 2016) and Vision Transformer (ViT) (Dosovitskiy et al., 2020) have demonstrated remarkable performance, surpassing even human discrimination capabilities.

**Figure 1:**
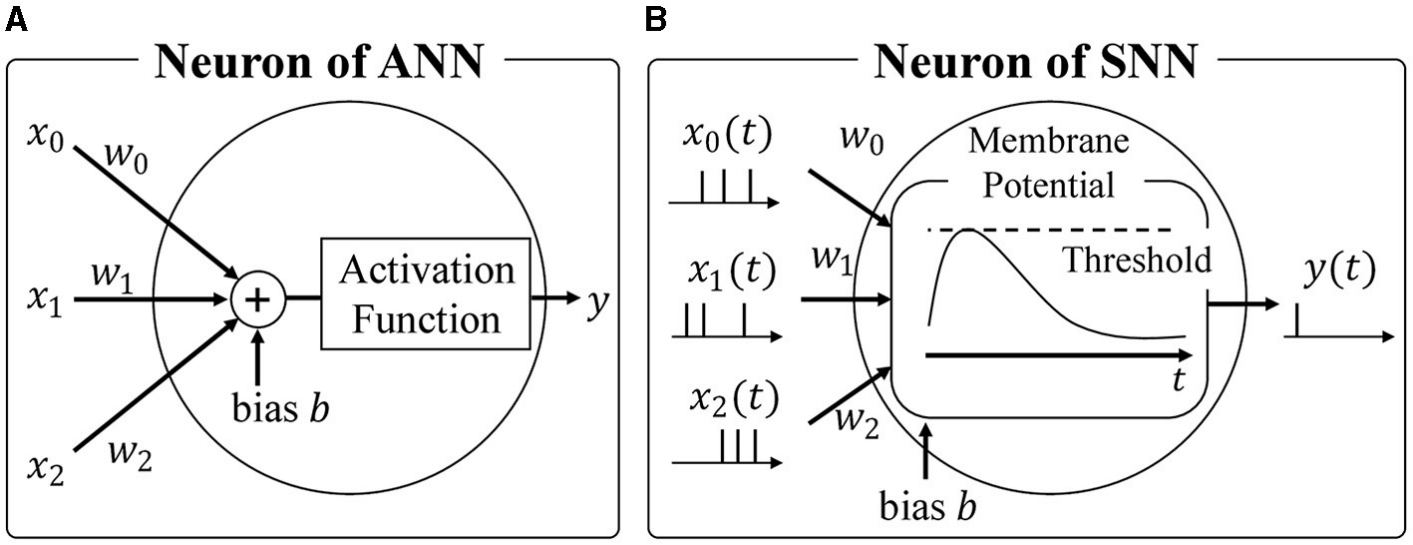
**(A)** Neuron of ANN, **(B)** Neuron of SNN.

Most widely used neural networks today are based on the formal neuron model, forming what is known as Artificial Neural Networks (ANNs) (Hopfield and Tank, 1985; Dong et al., 2021; Sharifani and Amini, 2023). As show in Figure 1A, ANNs employ the formal neuron model to calculate the weighted sum of inputs, followed by a non-linear activation function like ReLU or Sigmoid. Typically, activations and weights are represented as single-precision floating-point or 8-bit integer values. While the energy required for each multiplication may seem negligible at around 0.2 pJ per operation with 8-bit precision (Courbariaux et al., 2016), modern neural networks consist of millions of neurons, resulting in significant overall energy consumption during inference (Bernstein et al., 2021). Therefore, reducing the energy required for multiplications is crucial to minimize power consumption during neural network inference.

Spiking Neural Networks (SNNs) (Maass, 1997; Taherkhani et al., 2020; Nunes et al., 2022; Eshraghian et al., 2023) have gained attention as an alternative to ANNs. As shown in Figure 1B, SNNs emulate the biological brain's functionality, representing activations as spike trains comprising binary spike states (spike firing or absence of firing). This sparse spike representation offers two advantages when considering hardware accelerators. Firstly, the expensive integer or floating-point multiplications in ANNs can be replaced with additions. Unlike ANNs where activations are multiplied by synaptic weights, SNNs track changes in membrane potential by simply adding the synaptic weight upon receiving a spike event, as spikes are binary. Secondly, SNNs only require updating membrane potentials when they receive spikes, aligning well with asynchronous circuits and further reducing energy consumption. Leveraging the sparsity of spike events and event-driven computation, SNNs offer exceptional power efficiency, making them a preferred choice for neuromorphic architectures. Notably, IBM's TrueNorth (Akopyan et al., 2015) and Intel's Loihi (Davies et al., 2018) are hardware accelerators designed specifically for SNNs, successfully achieving significant energy reductions through asynchronous communication.

In addition to their low energy consumption, the learning algorithms for SNNs have dramatically improved in recent years. One such algorithm is the Surrogate Gradient (SG) method, which treats the non-differentiable spikes as differentiable smooth functions, allowing the SNN to be treated as a Recurrent Neural Network (RNN) and learned through Backpropagation through time (BPTT) algorithm. However, because the BPTT unfolds the SNN in the time direction for learning, the gradient propagates in the time direction, extending the propagation distance of the gradient. This can easily cause gradient vanishing/explosion, making it difficult to achieve sufficient inference performance in large-scale neural networks (Zenke and Vogels, 2021; Sun et al., 2022; Guo et al., 2023). Therefore, the ANN-SNN conversion, which maps the parameters learned by the ANN to the SNN, has been developed, making it possible to infer the SNN while maintaining the same accuracy as the ANN (Sengupta et al., 2019; Hu et al., 2021). The accuracy of models trained with each method using the CIFAR-10 dataset is shown in Table 1. From Table 1, it can be seen that when the parameters of the SNN are determined by the ANN-SNN conversion, inference performance comparable to that of the ANN can be achieved, but the performance obtained by the SG method is significantly inferior compared to the ANN. The ANN-SNN conversion is also used for complex tasks other than class classification, such as object detection. Kim et al. (2020) showed that by converting YoLo to SNN, an energy efficiency 280 times that of the ANN implementation can be achieved.

**Table 1:** Inference accuracy of models trained with SG and ANN-SNN conversion.

	**ANN**	**SG**	**ANN-SNN conversion**
VGG11	92.2%	86.3%	92.2%
VGG16	93.9%	80.8%	93.7%
VGG19	93.6%	71.6%	93.6%

Despite the superior characteristics of SNNs over ANNs, there is still potential for energy and latency reduction. SNNs rely on spike firing probabilities to represent information, necessitating sufficiently long spike firing sequences to accurately measure these probabilities.

In this paper, we propose a method to reduce inference energy and latency in SNNs based on Bayesian fusion. Spikes can be represented as binary variables indicating firing or non-firing states. The observed number of spike firings in a given time period can be modeled using a binomial distribution. In other words, decoding information represented by a spike firing sequence is equivalent to determining the most probable parameters of the binomial distribution that generated the observed spike firing sequence. In general, there is a trade-off between inference accuracy, latency, and energy consumption. Longer observation of spike firing sequences allows for higher accuracy in parameter estimation but increases latency and reduces energy efficiency. In this study, we reduce energy consumption and latency while maintaining inference accuracy by compensating for information degradation resulting from shortened spike sequence observations using prior knowledge about spike firing probabilities. Specifically, we predict the probability of firing for neurons in the final output layer based on the firing sequences of neurons in the shallow layers of the network. We employ Bayesian fusion with the firing sequences observed in the final output layer to reduce the required length of spike firing sequence observations without compromising the accuracy of firing probability estimation. Numerical experiments utilizing VGGs and ResNets demonstrate that we can achieve up to 48.0% energy reduction while maintaining inference accuracy in image classification tasks involving MNIST, CIFAR-10, and CIFAR-100 datasets.

## 2 Related works

### 2.1 Enhancing inference efficiency in SNN

Research focused on reducing the inference time steps of Spiking Neural Networks (SNNs) converted from Artificial Neural Networks (ANNs) is actively pursued (Hwang et al., 2021; Bu et al., 2023; Rathi and Roy, 2023). Various techniques for converting ANNs into efficiently inferable SNNs have been proposed, such as Robust Normalization (Rueckauer et al., 2017) RTS (Deng and Gu, 2021) and RMP (Han et al., 2020). Methods addressing the discrete spike sequences of SNNs include using clip functions or quantized ReLU functions instead of ReLU during pre-conversion training, as seen in TCL (Ho and Chang, 2021) and QCFS (Bu et al., 2023). Innovations applied directly to SNN neuron models, like the “reset-by-subtraction” method for resetting neuron firing potentials, minimize information loss compared to resetting methods that force the membrane potential to zero, thereby enabling faster inference (Diehl et al., 2015; Hwang et al., 2021; Rathi and Roy, 2023).

Apart from techniques for converting to efficiently inferable SNNs, strategies to enhance network structure for reducing inference time have also been proposed. For example, the early exit model outputs the final classification result from shallow layers without waiting for deeper layer results when input classification is straightforward (Chen et al., 2023; Li Y. et al., 2023). This model incorporates an internal classifier (IC) that predicts the final output from activations in internal layers of a multi-layer neural network (Li C. et al., 2023). In this approach, inference terminates early and outputs the final prediction once the confidence in predictions from the IC exceeds a predefined threshold.

Traditionally, methods have primarily focused on early termination or reduction of inference time in the final classifier layer, switching between early termination based on confidence in the IC or final classifier (FC), limiting accuracy to the precision of the IC or FC alone. Therefore, the proposed method achieves higher performance by statistically integrating outputs from both intermediate and final classifiers, surpassing what a single classifier can achieve.

### 2.2 Hardware accelerator for SNN

The sparse representation of spikes suits well with hardware, and a variety of SNN chips have been proposed, led by IBM's TrueNorth. TrueNorth is a highly specialized processor that can handle a specific model of spiking neurons (Akopyan et al., 2015). TrueNorth consists of 4,096 cores, each of which has a crossbar consisting of 256 axons and 256 neurons. The cores are connected to each other by a two-dimensional mesh network, and any neuron can be connected to any axon. In order to reduce the circuit size of the neurons, a virtual neuron scheme is adopted where a single neuron circuit is time-shared. Each neuron in TrueNorth uses an event-driven circuit that updates the membrane potential upon receiving a spike, which has succeeded in significantly reducing power consumption. For example, the TrueNorh chip, manufactured in a 28 nm process, uses 5.4 billion transistors, yet requires only 63 mW to recognize a 400 × 240 pixel image input at 30 FPS. The energy consumption per spike ignition is 26 pJ.

Another SNN accelerator is the SpiNNaker (Merolla et al., 2014) being developed at the University of Manchester, which consists of 18 ARM9s, a lightweight general-purpose processor core developed by ARM, and a dedicated processor that handles the interprocessor connections. In contrast to TrueNorth, which specializes in efficient simulation of integrate-and-fire models, SpiNNaker, which uses general-purpose processors, can run arbitrary neuron models. In addition, SpiNNaker can efficiently transfer spike information through multicast communication, and a single board with 48 chips can simulate a neural network of 250,000 neurons and 80 million synapses in real time.

Table 2 summarizes the differences between ANNs and SNNs.

**Table 2:** Comparison of ANN and SNN.

	**ANN**	**SNN**
Neuron model	Formal neuron model	IF Neuron model
Time-dependency	No	Yes
Data transmitted between neurons	Single-precision	Binary spikes
	Floating-point number	
	8-bit integers	
Representation of data	Activation	Spike firing
		Probability
Operation cost	The number of connections between neurons	Event-Driven (the number of spikes)
Hardware to execute	CPU	Neuromorphic
	GPU	Hardware
	TPU	(IBM TrueNorth et al.)

## 3 Preliminary

### 3.1 Artificial neural network

Each neuron in the ANN takes the product of the input activation *x* and the synaptic coupling weights *w*, and adds a bias *b*. This is then passed through a nonlinear function *f* to obtain the output activation *y* as follows Equation (1):


(1) $$\begin{array}{ll} y & =f\left(\overset{n}{\underset{i=1}{\sum }}w_{i}x_{i}+b\right). \end{array}$$

Activation values are often represented by single-precision or half-precision floating-point numbers, or by 8-bit integers.

In ANN, when the parameters of one layer change during training, the input distribution for the subsequent layers changes. Since this change increases as the layer depth increases, it is difficult to take a large learning rate in order to suppress learning divergence, which is known to be a “covariate shift” problem. To solve this problem, the Batch Normalization (BN) technique has been proposed (Ioffe and Szegedy, 2015). BN normalizes the input of the layers in each mini-batch to have mean 0 and variance 1, followed by scaling and biasing processes using learnable scaling factors and bias parameters. More specifically, the operation of BN layer is represented as follows Equation (2):


(2) $$\begin{array}{ll} y^{\prime } & =\gamma \frac{y-\mu }{\sqrt{\sigma^{2}+\epsilon }}+\beta , \end{array}$$

where μ and σ^2^ are the mean and variance for each mini-batch, respectively, and γ and β are the learnable scaling factor and bias parameter. Since μ, σ^2^, γ, and β are fixed and treated as constants during inference, the batch normalization layer can be fused into the previous linear layer during inference to reduce the number of operations. Specifically, they can be integrated into the weights of the previous layer, as shown in Equations (3, 4).


(3) $$\begin{array}{ll} \hat{w_{i}} & =\frac{\gamma }{\sigma }w_{i} \end{array}$$

(4) $$\begin{array}{ll} \hat{b} & =\frac{\gamma }{\sigma }\left(b-\mu \right)+\beta \end{array}$$

### 3.2 Spiking neural network

The biological brain is believed to represent information by transient voltage signals called spike firing, and a computational model that mimics this mechanism is called the spiking neural network (SNN). In this study, we use the integrate-and-fire (IF) model, which is considered to be the most popular model and has been proposed in many hardware implementations. In the IF model, a neuron is represented as a node with a membrane potential as its internal state. When a neuron receives a spike from another neuron, it updates its membrane potential according to the synaptic connection weights between it and that neuron. This behavior can be described as follows:


(5) $$\begin{array}{ll} V_{i}^{t} & =V_{i}^{t-1}+\underset{j}{\sum }w_{ij}V_{th}\Theta_{j}^{t}+b_{i}, \end{array}$$

where $V_{i}^{t}$ is the membrane potential of neuron *i* at time *t*, *w*_*ij*_ is the synaptic weight from *i*-th neuron to *j*-th neuron, *V*_*th*_ is the threshold voltage, and *b*_*i*_ is the bias value of the *i*-th neuron. $\Theta_{j}^{t}$ is a binary variable that represents the presence or absence of spike firing of *j*-th neuron at time *t*. This is a binary variable that represents the presence or absence of spike firing in *j*-th neuron at time *t*, and is calculated from the membrane potential of *j*-th neuron as follows:


(6) $$\begin{array}{ll} \Theta_{j}^{t} & =\{\begin{array}{ll} 1 & V_{j}^{t}>V_{th}\\ 0 & \text{otherwise}. \end{array} \end{array}$$

Each neuron resets its membrane potential after firing a spike. There are two methods of resetting the membrane potential: setting the membrane potential to zero or subtracting the threshold voltage. The latter method is known to cause less information degradation (Rueckauer et al., 2017), so we adopt the latter method in this study. The method is described as follows:


(7) $$\begin{array}{ll} V_{i}^{t} & =V_{i}^{t}-V_{th}\Theta_{i}^{t}. \end{array}$$

Combining Equations (5–7), we can derive an update rule for the membrane potential of the *i*th neuron in the *l*th layer as:


(8) $$\begin{array}{ll} V_{l,i}^{t} & =V_{l,i}^{t-1}+\underset{j}{\sum }w_{ij}V_{th}\Theta_{l-1,j}^{t}+b_{i}-V_{th}\Theta_{l,i}^{t}. \end{array}$$

### 3.3 ANN-to-SNN conversion

While information transfer using binary spikes greatly improves the energy efficiency of SNNs, it also makes learning by backpropagation, which requires gradient computation, difficult. There is some research using the STDP rule, which changes the synaptic connection weights according to the time difference between spikes, which is considered to be one of the basic learning algorithms of the biological brain (Bi and Poo, 1998). However, its application is limited to simple tasks such as MNIST, and it is still difficult to perform very complex tasks such as those realized by modern DNNs (Diehl and Cook, 2015). To solve this problem, a method was proposed to convert the weights learned in the ANN to SNNs and only perform inference in SNNs (Rueckauer et al., 2017). The basic principle of converting ANNs into SNNs is to match the output activity value of ReLU with the firing rate of spiking neurons. To obtain the conversion equation from ANN to SNN, we first accumulate (Equation 8) over the simulation timestep from time 1 to *T*, divide both sides of the equation by *T*, and yield Equation (9):


(9) $$\begin{array}{ll} \frac{V_{l,i}^{t}}{T} & =\frac{V_{l,i}^{0}}{T}+\overset{N}{\underset{j=1}{\sum }}w_{ij}\overset{T}{\underset{t=1}{\sum }}\frac{V_{th}\Theta_{l-1,j}^{t}}{T}+b_{i}-V_{th}\overset{T}{\underset{t=1}{\sum }}\frac{\Theta_{l,i}^{t}}{T}. \end{array}$$

Let $p_{l,i}=\overset{T}{\underset{t=1}{\sum }}\Theta_{l,i}^{t}/T$ be the spike firing probability of the *i*-th neuron in the *l*-th layer, and written as:


(10) $$\begin{array}{l} p_{l,i}=\frac{1}{V_{th}}\left(\overset{N}{\underset{j=1}{\sum }}w_{i,j}V_{th}p_{l-1,j}+b_{i}-\frac{V_{l,i}^{t}-V_{l,i}^{0}}{T}\right). \end{array}$$

From Equation (10), it can be inferred that the spike firing rate is proportional to the weighted sum of the input spike firing rate, excluding $\left(V_{l,i}^{t}-V_{l,i}^{0}\right)/T$. Note that the membrane potential has an initial value at *t* = 0, whereas spikes are observed starting from *t* = 1.

## 4 Error analysis of converted SNN

In order to improve the energy efficiency of SNNs, it is important to first analyze the error factors in detail. To this end, we firstly classify the error factors into two types: errors that are incurred during the ANN to SNN conversion process and those incurred when decoding the spike firing representation of the information.

### 4.1 Errors induced during ANN to SNN conversion process

The error *E*_*l, i*_ in the ANN-SNN conversion can be calculated as the difference between the activation value *y*_*l, i*_ of the ANN and the firing rate *V*_*th*_*p*_*l, i*_ of the SNN scaled by the threshold, i.e., *E*_*l, i*_ = *y*_*l, i*_−*V*_*th*_*p*_*l, i*_. The application of the ANN-SNN conversion assumes the use of the ReLU function as the activation function of the ANN, and *E*_*l, i*_ is given by Equation (11):


(11) $$\begin{array}{l} E_{l,i}\;=\text{ ReLU}\left(\sum_{j=1}^{n}w_{i,j}y_{l-1,j}+b_{i}\right)\\ \;-\left(\sum_{j=1}^{N}w_{i,j}V_{th}p_{l-1,j}+b_{i}-\frac{V_{l,i}^{t}-V_{i,i}^{0}}{T}\right) \end{array}$$

where *y*_*l, i*_ represents the activation value of neuron *i* in layer *l* and *T* represents a simulation timestep, which is a positive integer. If the input to the ReLU function, $\overset{n}{\underset{j=1}{\sum }}w_{i,j}y_{l-1,j}+b_{i}$, is positive, *E*_*l, i*_ is given by


(12) $$\begin{array}{l} E_{l,i}=\overset{N}{\underset{j=1}{\sum }}w_{i,j}\left(y_{l-1,j}-V_{th}p_{l-1,j}\right)+\frac{V_{l,i}^{t}-V_{l,i}^{0}}{T}. \end{array}$$

Noting that *y*_*l*−1, *j*_−*V*_*th*_*p*_*l*−1, *j*_ = *E*_*l*−1, *j*_, this equation can be further written as Equation (13):


(13) $$\begin{array}{l} E_{l,i}=\overset{N}{\underset{j=1}{\sum }}w_{i,j}E_{l-1,j}+\frac{V_{l,i}^{t}-V_{l,i}^{0}}{T}. \end{array}$$

This shows that the error in the activation values of the ANN and SNN at layer *l* is the sum of the weighted errors at layer *l*−1 plus $\left(V_{l,i}^{t}-V_{l,i}^{0}\right)/T$. On the other hand, if the input to the ReLU function, $\overset{n}{\underset{j=1}{\sum }}w_{i,j}y_{l-1,j}+b_{i}$, is negative, the neuron does not fire, *p*_*l, i*_ = 0, and therefore *E*_*l, i*_ = 0.

The error *E*_1, *i*_ at the input layer depends on the coding scheme of the input. In SNNs, a method called direct coding, which directly uses floating point values as the input to the first layer, is common, and in this case, *E*_1, *i*_ = 0 (Rathi and Roy, 2023). Conventionally, the membrane potential $V_{l,i}^{0}$ of the SNN is initialized to 0 and the threshold *V*_*th*_ is fixed at 1, so Equations (10, 12) can be simplified as follows Equations (14, 15):


(14) $$\begin{array}{ll} p_{l,i} & =\overset{N}{\underset{j=1}{\sum }}w_{i,j}p_{l-1,j}+b_{i}-\frac{V_{l,i}^{t}}{T} \end{array}$$

(15) $$\begin{array}{ll} E_{l,i} & =\overset{N}{\underset{j=1}{\sum }}\left(y_{l-1,j}-p_{l-1,j}\right)+\frac{V_{l,i}^{t}}{T}. \end{array}$$

In the following, we assume that the membrane potential $V_{l,i}^{0}$ is initialized to 0 and the threshold *V*_*th*_ is fixed at 1.

According to Equation (15), we notice that the conversion of ANN to SNN induces an error term $V_{l,i}^{\left(t\right)}/T$, which is inversely proportional to the integration time *T*. Although increasing *T* will decrease the estimation error, it will also increase the energy required for inference. Hence, there is a trade-off between inference accuracy and energy. We also notice that the spike firing probability *p*_*l, i*_ is restricted to a range of [0, 1], whereas ANNs typically have no such constraints. For instance, if the threshold *V*_*th*_ is extremely high compared to the synaptic weights, it takes a long time for the membrane potential to reach *V*_*th*_, resulting in a low spike firing probability. Conversely, if *V*_*th*_ is extremely small compared to the synaptic weight, the membrane potential will exceed *V*_*th*_ regardless of the spike input, which again causes information degradation.

Hence, synaptic weight *W*_*l, i, j*_ should be carefully normalized to avoid too low or too high spike firing probability. To this end, various data-driven normalization methods have been proposed (Cao et al., 2015; Diehl et al., 2015). One of the well-known methods is “layer-wise normalization” proposed by Diehl et al. (2015), where the synaptic weights are normalized so that the maximum activations calculated using the training dataset does not exceed *V*_*th*_ (i.e. 1.0). Hence, the synaptic weights $w_{l}^{\text{SNN}}$ are calculated as follows:


(16) $$\begin{array}{ll} w_{l}^{SNN} & =\frac{\lambda_{l-1}}{\lambda_{l}}w_{l},b_{l}^{SNN}=\frac{1}{\lambda_{l}}b_{l}, \end{array}$$

where λ_*l*_ is the maximum activations in *l*-th layer calculated by using the training dataset. Later, a modified layer-wise normalization has been proposed, where λ_*l*_ is selected to be 99.9th percentile of the maximum activations to improve the robustness to outliers (Rueckauer et al., 2017). More recently, Kim et al. have proposed “channel-wise normalization” (Kim et al., 2020). In addition to this normalization, methods for reducing errors by adjusting the threshold have also been developed (Sengupta et al., 2019; Park et al., 2020).

Furthermore, techniques have been proposed to reduce SNN errors by pre-charging the initial membrane potential to promote early firing of the first spike (Hwang et al., 2021). Bu et al. (2023) demonstrated that neurons fire more uniformly by using floor and clipping functions instead of the ReLU function during ANN training and initializing the membrane potential to half of the threshold during SNN inference.

### 4.2 Error induced during decoding spike outputs

Since SNNs represent information in terms of spike firing frequency, the inference results of SNNs need to be decoded again into a continuous value representation. Let a spike train of an output neuron be Θ = (Θ^(1)^, Θ^(2)^, ⋯ , Θ^(*T*)^). Since Θ^(*t*)^ can be assumed to follow a Bernoulli distribution, its probability function can be modeled by the following Bernoulli distribution:


(17) $$\begin{array}{ll} P\left(\Theta^{\left(t\right)}|p\right) & =p^{\Theta^{\left(t\right)}}{\left(1-p\right)}^{1-\Theta^{\left(t\right)}}. \end{array}$$

The conventional approach to recover original ANN activations from the spike trains Θ is based on *maximum likelihood estimation* (MLE). Hence, finding the spike firing probability is equivalent to finding the parameters of Bernoulli distribution, i.e. *p*, so that the probability of observing **Θ** is maximized. The likelihood of observing **Θ** is equal to:


(18) $$\begin{array}{ll} P\left(\Theta |p\right) & =\overset{T}{\underset{t=1}{\prod }}P\left(\Theta^{\left(t\right)}|p\right), \end{array}$$

where *T* is observation time. Substituting Equation (17) into Equation (18), we have:


(19) $$\begin{array}{ll} P\left(\Theta |p\right) & =p^{M}{\left(1-p\right)}^{T-M}, \end{array}$$

where *M* is the number of spike firings observed and is given by $M=\overset{T}{\underset{i=1}{\sum }}\Theta^{\left(t\right)}$. Our objective here is to find *p* that maximizes Equation (19) given a spike train Θ. To this end, we first take logarithm of Equation (19) and obtain:


(20) $$\begin{array}{l} \log\left[P\left(\Theta |p\right)\right]=M\log p+\left(T-M\right)\log\left(1-p\right). \end{array}$$

Since logarithm is a monotonically increasing function, maximizing Equation (20) is equivalent to maximizing Equation (19). Hence, the gradient of log[*p*(Θ|*p*] should be zero at the optimal *p*_*opt*_ as Equation (21):


(21) $$\begin{array}{l} \frac{\partial }{\partial p}\log\left[P\left(\Theta |p\right)\right]{|}_{p=p_{opt}}=\frac{M}{p_{opt}}-\frac{T-M}{1-p_{opt}}=0. \end{array}$$

**Figure 2:**
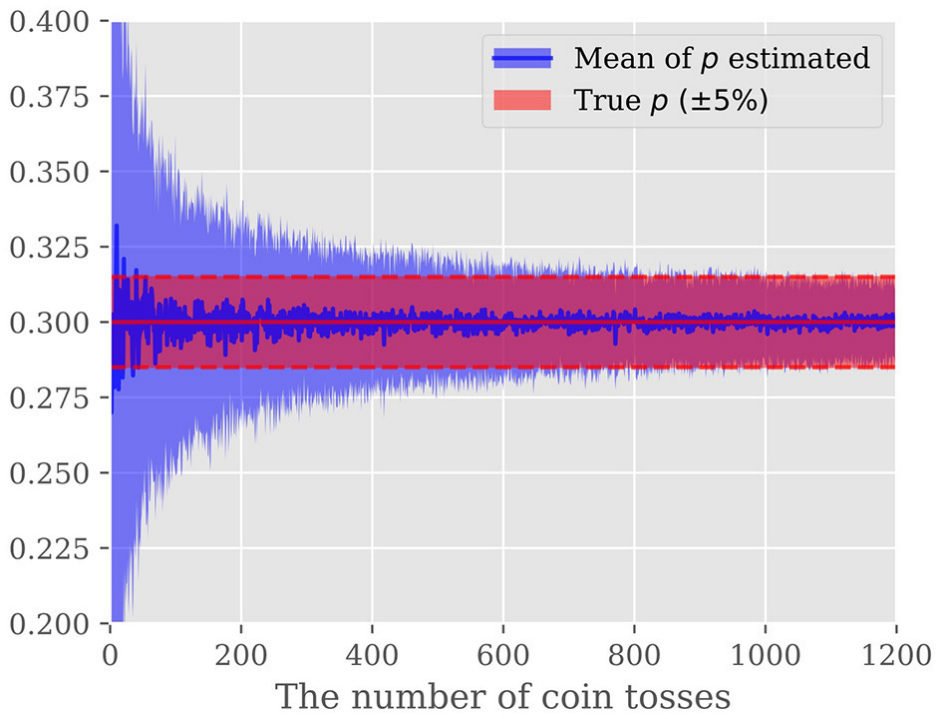
Predicted probability that the coin will turn up from the result of the coin toss: The vertical axis shows the probability and the horizontal axis shows the number of times the coin was tossed. The blue line shows the mean of the predicted probabilities, and the light blue area shows the standard deviation of the probabilities. The red area indicates the region within a 5% error of the golden probability of 0.3.

Although MLE results in an unbiased estimation of *p* without relying on any prior knowledge, it frequently suffers from degraded estimation accuracy. Let us take an unfair coin toss example, where the unknown probability of head, *p*, is estimated given a sequence of heads and tails resulting from tossing an unfair coin *N* times. Figure 2 shows the estimated probability of head *p* as a function of a number of coin-tosses *N*_*ex*_. The horizontal line shows the golden probability of 0.3. To show the randomness, the same experiment is repeated 100 times. We notice that to estimate the unknown probability *p* within 5% accuracy, approximately 1000 times coin tosses are required.

As described above, the SNN converted from the ANN expresses values based on spike firing probabilities, so there is a trade-off between the accuracy and the period of time that spike firings are observed. In other words, if spike firing can be observed for a long enough period of time, the firing probability can be estimated with high accuracy, but the energy and latency will increase. Thus, this study improves this trade-off by incorporating Bayesian methods into the estimation of spike firing probabilities.

## 5 Methods

As we saw in the previous section, MLE-based estimation requires hundreds of spikes to be observed for estimating the spike firing probability with an acceptable accuracy. To alleviate this problem, we propose to incorporate the prior knowledge of the spike firing probability with the observed spike train to improve the estimation accuracy. Our proposal is based on the observation that the activations of neurons at an early layer of DNN may often carry sufficient information for predicting activations of neurons in the final layer (Teerapittayanon et al., 2016). Hence, by exploiting the spike firing probability of neurons at an early layer as prior knowledge, we may compensate for the information degradation caused by reducing the number of spikes to be observed. In the following, we firstly derive the detailed formulation of BayesianSpikeFusion and demonstrate its effectiveness compared with MLE using the coin toss example again. Then, we provide the method to transform the early layer activations into the prior knowledge. Finally, the detailed algorithm is provided.

### 5.1 Definition of prior distribution

To formulate the spike firing probability estimation problem with the Bayesian model, we have to firstly find a way to encode the prior knowledge of the spike firing probability. To this end, we exploit Beta distribution whose probability density function (PDF) is given by:


(22) $$\begin{array}{ll} p\left(p|\alpha ,\beta \right) & =\frac{p^{\alpha -1}{\left(1-p\right)}^{\beta -1}}{B\left(\alpha ,\beta \right)}, \end{array}$$

where *B*(·, ·) is Beta function defined as Equation (23):


(23) $$\begin{array}{ll} B\left(x,y\right) & =\int_{0}^{1}t^{x-1}{\left(1-t\right)}^{y-1}dt. \end{array}$$

**Figure 3:**
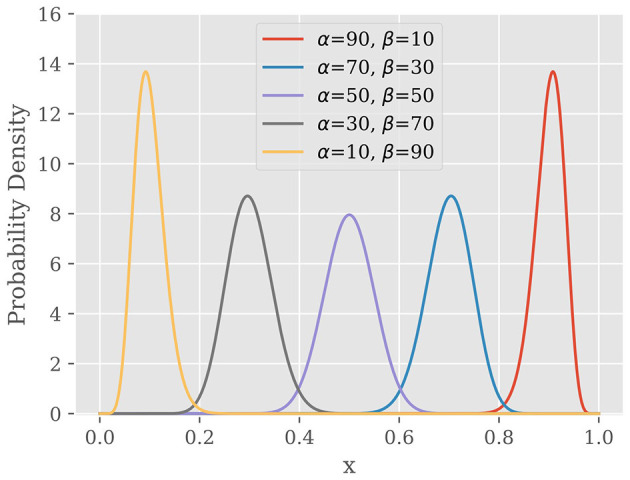
Probability density function of Beta distribution.

Figure 3 shows the probability density function of Beta distribution with different values of α and β, which reveals three important properties of Beta distribution. First, Beta distribution is defined over [0, 1] which covers all possible spike firing probability. Second, the PDF of Beta distribution has a peak at a particular value which is called as “mode” of distribution. Third, when one of α and β becomes larger, the peak is biased toward the edge.

Suppose *p*_*prior*_ be the spike firing probability estimated from the activations of neurons at an early layer. To encode the prior knowledge, the prior distribution should have peaked at *p*_*prior*_. Hence, we set up the following constraint for the hyperparameter α and β:


(24) $$\begin{array}{ll} p_{prior} & =\frac{\alpha -1}{\alpha +\beta -2}. \end{array}$$

Equation (24) can be rewritten as:


(25) $$\begin{array}{ll} \beta & =\frac{\left(1-p_{prior}\right)\left(\alpha -1\right)}{p_{prior}}+1. \end{array}$$

Substituting Equation (25) into Equation (22), we have:


(26) $$\begin{array}{ll} P\left(p|\alpha \right) & =\frac{p^{\alpha -1}{\left(1-p\right)}^{\frac{\left(1-p_{prior}\right)\left(\alpha -1\right)}{p_{prior}}}}{B\left(\alpha ,\frac{\left(1-p_{prior}\right)\left(\alpha -1\right)}{p_{prior}}+1\right)}. \end{array}$$

In Equation (26), there is only one hyper-parameter α. The selection of α will be discussed in detail in Section 5.4.

### 5.2 Bayesian estimation of spike firing probability

With the prior distribution defined, we can now model the posterior distribution for μ, which represents our belief about μ after obtaining the spike train Θ as follows:


(27) $$\begin{array}{ll} P\left(p|\Theta ,\alpha \right) & \propto P\left(\Theta |p\right)P\left(p|\alpha \right). \end{array}$$

Here, we applied Bayes' theorem to derive the right from the left side. Substituting Equations (19, 26) into Equation (27), we have:


(28) $$\begin{array}{ll} P\left(p|\Theta \right) & =\frac{p^{M+\alpha -1}{\left(1-p\right)}^{N-M+\frac{\left(1-p_{prior}\right)\left(\alpha -1\right)}{p_{prior}}}}{B\left(M+\alpha ,N-M+\frac{\left(1-p_{prior}\right)\left(\alpha -1\right)}{p_{prior}}+1\right)}. \end{array}$$

Since the posterior distribution represents our belief about *p* after observing the spike train Θ, our goal is to find the value of *p*_MAP_ at which the posterior distribution *P*(*p*|Θ) is maximized. Note here that the posterior distribution *P*(*p*|Θ) in Equation (28) is in the same probability density family as the prior distribution *P*(*p*|α) in Equation (26) and as we examined in Figure 3, the posterior distribution has peak at a certain *p*. Hence, *p*_MAP_ is given by the mode of the Beta distribution in Equation (29) as follows:


(29) $$\begin{array}{ll} p_{\text{MAP}} & =\frac{M_{l,i}+\alpha -1}{N+\left(\alpha -1\right)/r}. \end{array}$$

**Figure 4:**
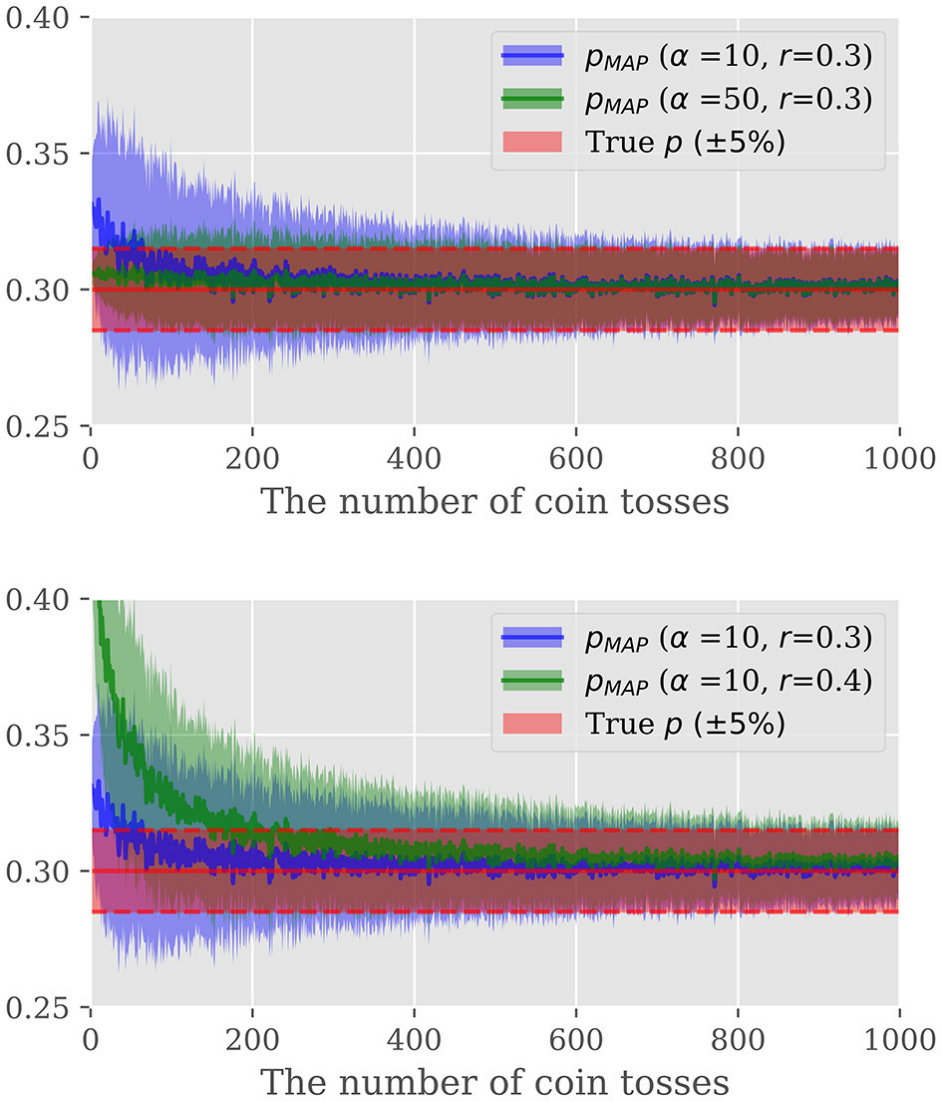
**Top**: Comparison of the effect of α, **Bottom**: Comparison of the effect of *r*. The vertical axis shows the probability and the horizontal axis shows the number of times the coin was tossed. The blue and green lines show the change in the Bayesian estimated probabilities with the parameters described in the legend. The red area indicates the region within 5% error of the golden probability of 0.3.

We here demonstrate the power of Bayesian estimation for estimating unknown probability of occurrence only given a certain length of observation. To this end, let us take the unfair coin toss example again. Figure 4 illustrates the MAP estimation result as a function of the observed trials. We repeated the same experiment for several α and *r* configurations to investigate the impact of a hyper-parameter and the mode of the prior distribution on the estimation. Figure 4 gives two important observations. First, the prior distribution has a large effect when the sample size is small, whilst as the sample size increases, the likelihood of Equation (19) becomes dominant. Hence, with properly selected prior distribution, we can safely reduce the number of spikes to be observed without deteriorating the estimation accuracy. Second, the hyper-parameter α controls the strength of the prior belief; large α biases the estimation toward the prior distribution while the smaller α results in the estimation put more emphasis on the observation. Hence, the selection of α is the integral part to improve the efficiency. In the followings, we firstly consider the construction of the prior distribution, followed by the hyper-parameter selection.

### 5.3 How to build a prior distribution

The effectiveness of Bayesian fusion depends on the design of the prior distribution. If an appropriate prior distribution can be set, Bayesian fusion is very effective in improving the convergence speed, whereas an inappropriate prior distribution can hinder convergence. To this end, we exploit internal classifiers that have been presented in several works (Kaya et al., 2019).

**Figure 5:**
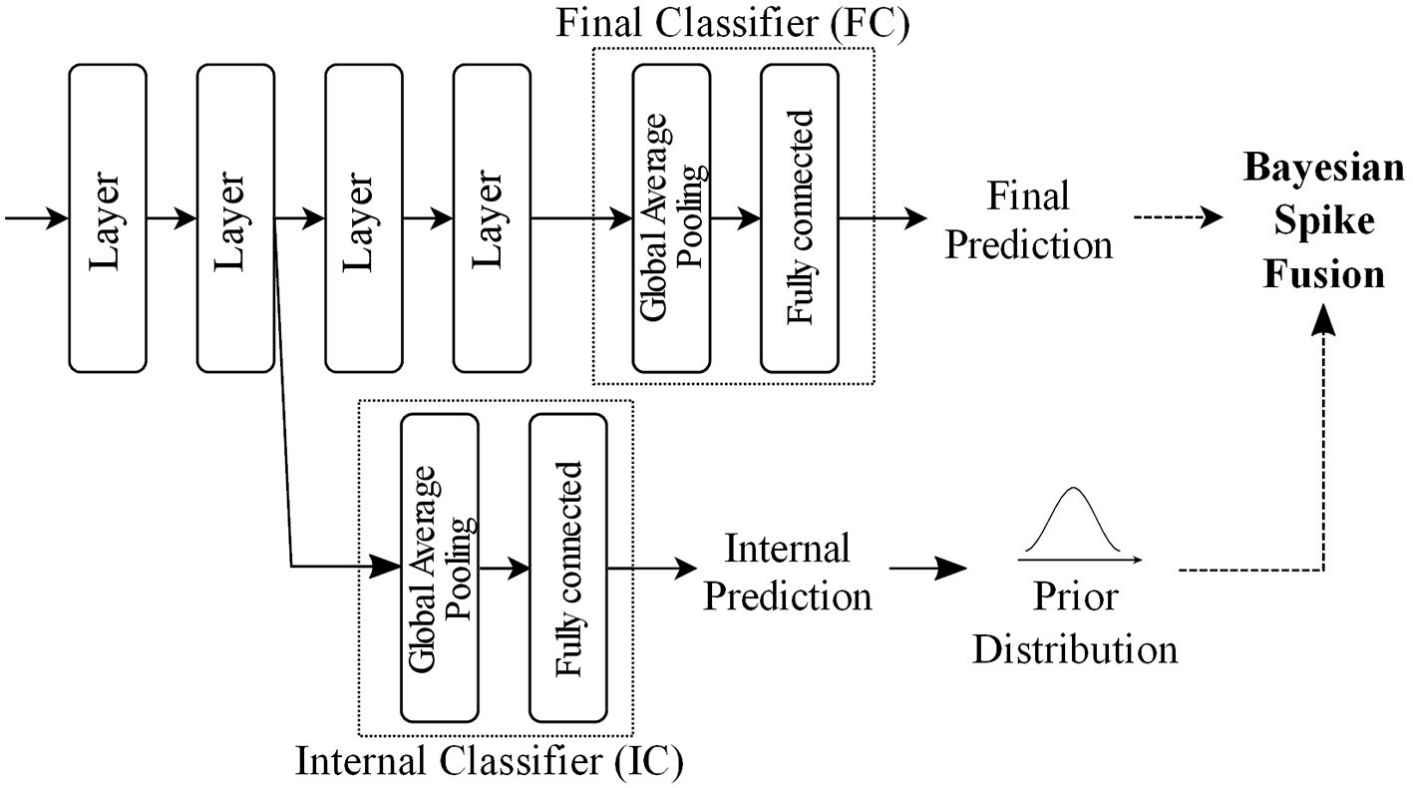
Example of BayesianSpikeFusion outline in a network with Internal Classifier (IC).

Figure 5 outlines an example BayesianSpikeFusion which is essentially a spiking neural network which includes a final classifier (FC) and an internal classifier (IC). The IC is strategically integrated into the network at a specific location, allowing it to predict both the activations of the output layer and the FC. The IC consists of two parts: a feature reduction layer and a fully connected layer to produce internal predictions. Although there are several possible approaches for implementing the reduction layer, this paper opts for Global Average Pooling (GAP) due to its simplicity of implementation and relatively small computational overhead. After the GAP, a fully connected layer is placed to yield early prediction. Specifically, in the proposed method, *p*_*prior*_ is set to be the average spike firing probability of neurons at the fully connected layer.

Our empirical evaluations reveal that IC accounts for only 11% of the whole computational workloads.

### 5.4 Hyper-parameter selection

As we saw in the previous section, the hyper-parameter α controls the strength of our belief over *r*; when α is large, the prior knowledge is expected to be accurate, and hence the prior distribution *P*(*r*|α) has a narrow peak around its mode *r*. Since the accuracy of the prior knowledge may differ from case to case, it must be adaptively tuned based on the observed spike trains. To this end, we employ “Empirical Bayes Method” and “Grid Search Method.”

**Empirical Bayes Method:** This method selects the hyperparameter α to maximize the marginal likelihood concerning *p* as follows Equation (30):


(30) $$\begin{array}{l} \alpha \;=\underset{\alpha }{\max}\int_{0}^{1}P(\Theta |p)P(p|\alpha )dp\\ \;=\underset{\alpha }{\max}\frac{B\left(M+\alpha ,N-M+\frac{\left(1-p_{prior})(\alpha -1\right)}{p_{prior}}+1\right)}{B\left(\alpha ,\frac{\left(1-p_{prior})(\alpha -1\right)}{p_{prior}}+1\right)}. \end{array}$$

**Figure 6:**
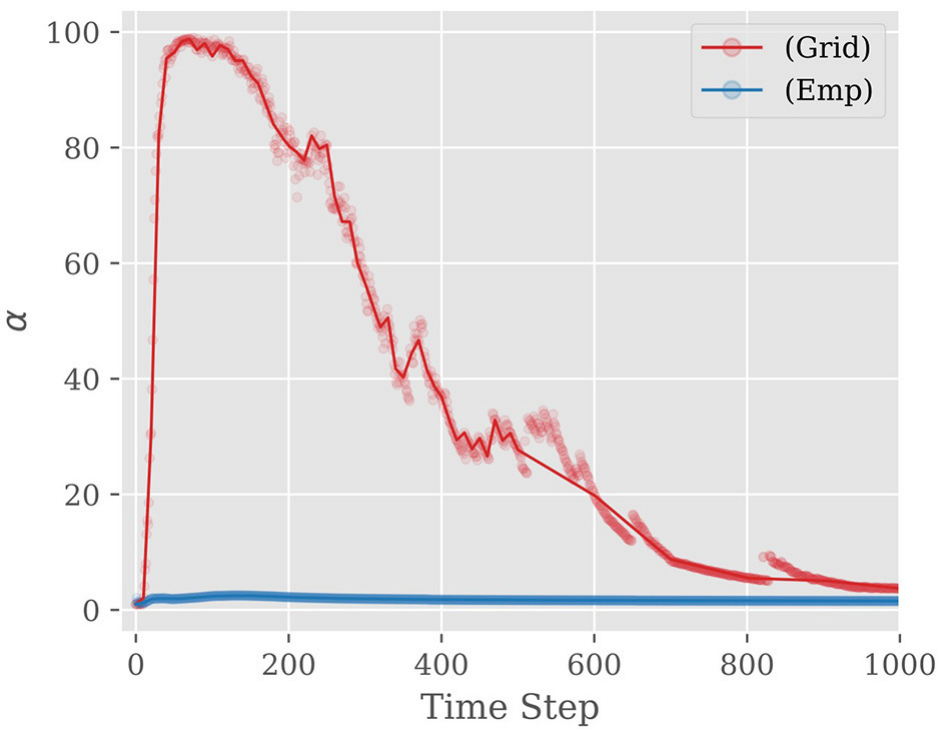
Examples of α and approximate curves when using VGG19 and CIFAR-10: (Grid) indicates Grid Search Method. (Emp) indicates Empirical Bayes Method. The value of α rises steeply until about the first 100 steps. Then, the value drops to around 0 before reaching 500 steps and finally converges to about 0.

**Grid search method:** Utilizing a subset of the training data grid search is conducted to determine the optimal value of α at time *t*. Specifically, commencing at *t* = 10 and progressing in increments of 10 up to 1,000, the α value that maximizes the classification accuracy on the subset of training data is selected. Figure 6 compares α selected by empirical bayes (Emp) and grid search (Grid) methods as a function of time step. Notably, it is observed that the optimal α selected by the grid search method tends to be initially large during the early stages of sampling and subsequently decreases as time step increase. This behavior can be attributed to the fact that when the time step is small, the spike propagation to deeper layers is insufficient, so the information from the IC located in the shallow layer is given more weight for inference accuracy. As the time step increases, the accuracy of the FC becomes higher, and hence, the weight assigned to the IC should be reduced for better performance. To reduce memory usage, the proposed method approximates the optimal α using a piecewise linear function, storing only the fitting parameters.

### 5.5 ANN training

The IC and the FC are trained simultaneously. However, when attempting to minimize the loss of the IC, the network weights tend to specialize in classifying at the IC, which compromises the accuracy of the FC. Therefore, this study focuses on training the network parameters to minimize a combined loss function that takes into account both the IC and the FC. Let the loss of the IC and the FC as *Loss*^*IC*^ and *Loss*^*FC*^, respectively. The overall loss function of the network is then defined as Equation (31):


(31) $$\begin{array}{ll} Loss & =Loss^{FC}+\tau \left(e\right)\cdot Loss^{IC}, \end{array}$$

where *e* represents the epoch, and τ(*e*) is a weighting coefficient that determines the emphasis on either *Loss*^*FC*^ or *Loss*^*IC*^ during training. Specifically, τ(*e*) is defined as Equation (32):


(32) $$\begin{array}{ll} \tau \left(e\right) & =\frac{N_{\text{MAC}}^{IC}}{N_{\text{MAC}}^{FC}}\cdot \frac{e}{N_{epoch}}, \end{array}$$

where $N_{\text{MAC}}^{IC}$ and $N_{\text{MAC}}^{FC}$ represent the number of multiply-accumulate (MAC) operations required for the forward propagation from the input to the IC and from the input to the FC, respectively. *N*_*epoch*_ denotes the total number of training epochs. Hence, at the beginning of the training, the network primarily focuses on minimizing the classification loss in FC. This allows the network to acquire effective intermediate representations for classification purposes. As the training progresses, the weight of the classification loss in IC is gradually increased. This adjustment enables the network parameters to be learned in such a way that the IC can accurately classify using the intermediate representations obtained during training.

## 6 Experiment

### 6.1 Experimental setting

In order to assess the effectiveness of the proposed approach, a numerical experiment is performed using PyTorch. The experimental setup involves utilizing networks from the VGGNet (VGG11, VGG16, and VGG19) and ResNet (ResNet18 and ResNet34) families. The experiment entails inserting IC after the ConvBlock or ResBlock, which comprises convolutional layers and Batch Normalize layers. The network is initially implemented and trained in the ANN domain. Subsequently, the trained weights are transformed for SNNs to evaluate the inference accuracy and the energy consumption of the entire network, including the Shallow Networks.

The target datasets are MNIST (LeCun et al., 1998), CIFAR-10, and CIFAR-100 (Krizhevsky and Hinton, 2009). MNIST consists of black-and-white images of handwritten digits from 0 to 9, 60k training images, 10k validation images, and their labels. CIFAR-10 and CIFAR-100 are 10- or 100-class image classification datasets for animals, plants, vehicles, etc., consisting of 50k training images and 10k validation images, and their labels.

The programming language used was Python 3.8, with PyTorch 1.12 as the machine learning library. The hardware accelerator used was NVIDIA RTX A6000, and the version of CUDA was 11.6.

### 6.2 Training of ANN

When training an ANN, we use Xavier's initialization method for the initial values of the weights (Glorot and Bengio, 2010). The input images were normalized with mean (0.5071, 0.4865, and 0.4409) and standard deviation (0.2673, 0.2564, and 0.2762) for each channel. An augmentation consisting of randomly cropping a 32 × 32 image after inserting 4 pixels of padding around it, flipping it left to right with probability 0.5, and randomly rotating it up to 15 degrees was performed. The number of training epochs was set to 120 epochs.

### 6.3 Conversion from ANN to SNN and inference by SNN

The trained weights are transformed for SNN by using Equation (16) with λ set to be the value at 99.9% of the activation when 5k images randomly selected from the training data are propagated through the network. In this experiment, we initialized all membrane potentials to 0 and set the threshold to 1. We also used 3,000 time steps for simulation and performed membrane potential resetting by subtraction.

In our energy calculations, we utilized SpikeSim, a simulator for SNN hardware accelerators based on in-memory computing (Moitra et al., 2023). SpikeSim employs multiple Processing Elements (PEs) that combine spikes and synaptic weights. These PEs form the Tile module, which integrates the accumulation module, the neuron module responsible for storing membrane potentials and comparing them to thresholds, the pooling module, and the global buffer that handles storage. These modules are interconnected in a mesh topology.

Each analog crossbar in SpikeSim consists of rows that receive spikes as potentials (*V*_*i*_) and columns that output the weighted sum of spikes as values (*I*_*j*_). RRAM devices are placed at the intersections of rows and columns. The conductance (*G*_*i, j*_) of these RRAMs is adjusted to match the learned synaptic weights. Input spikes flow through these RRAM devices, following Ohm's law, resulting in weighted currents. Additionally, currents generated from each row are summed according to Kirchhoff's law. These current values are converted to digital values through analog-to-digital conversion. The digitized values are aggregated in the accumulation module to compute membrane potentials. Finally, membrane potentials are sent to the neuron module, where they are compared to a threshold to determine spike firing.

SpikeSim implements each module as synchronous circuits, and in our study, we set the clock frequency to 250 MHz. The energy consumption calculated by SpikeSim includes the energy used for analog crossbar multiplication, other modules like the neuron module, and inter-module communication.

### 6.4 Experimental results

**Figure 7:**
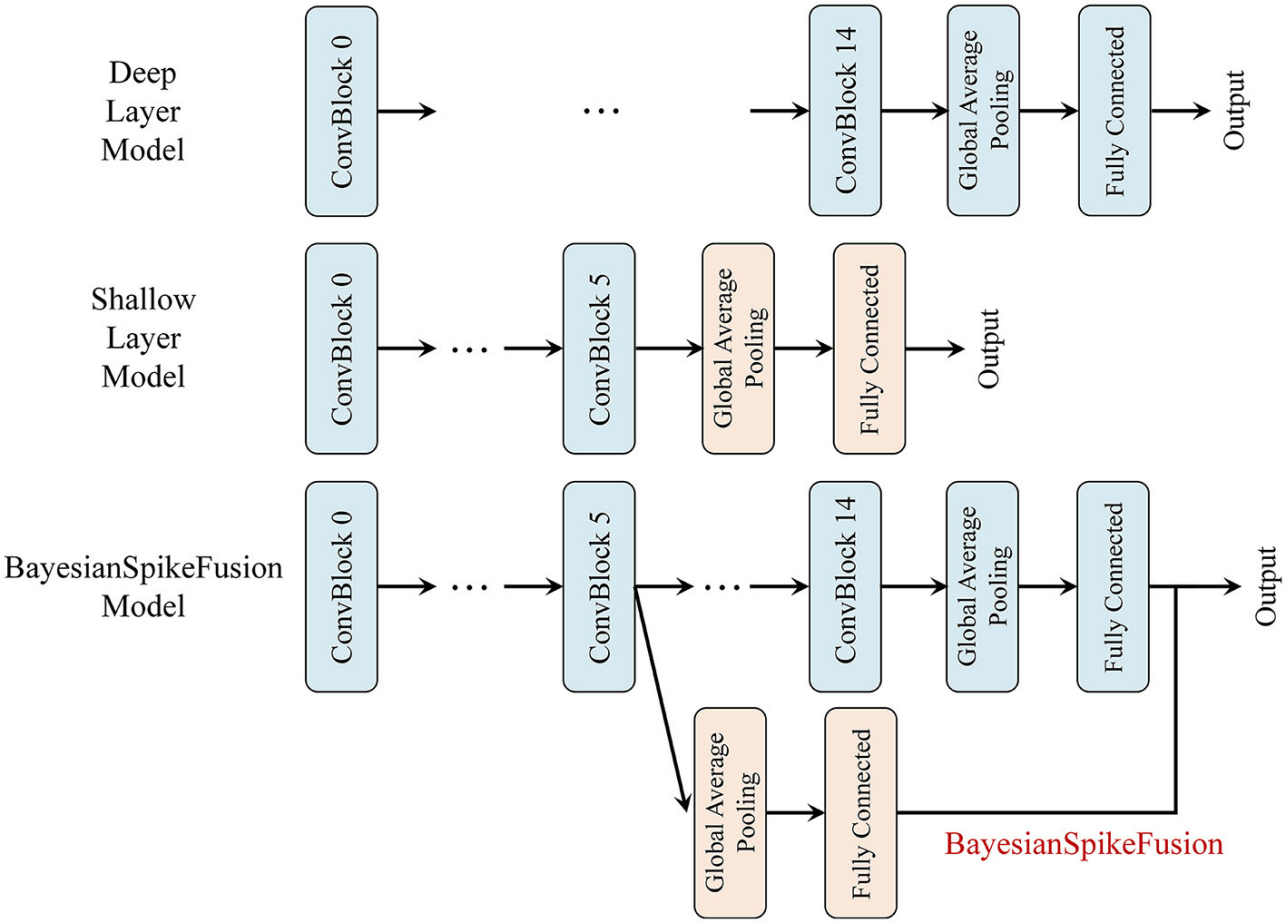
VGG19 network variants compared. **Top**: VGG19(DL), **Middle**: VGG19(SL), **Bottom**: BayesianSpikeFusion.

Throughout the experiment, we compare three models: a deep layer model (DL), an SNN converted from a corresponding ANN, prior to the incorporation of the IC, a shallow layer model (SL), a model that is composed of sub-network of (DL), i.e., from the input to the IC, with subsequent layers removed to form a shallow model, and (BayesianSpikeFusion) the proposed method that combines the IC and FC output with a Bayesian approach to obtain the final class classification outcome. Figure 7 visually depicts the three models with the IC inserted at the sixth layer.

**Figure 8:**
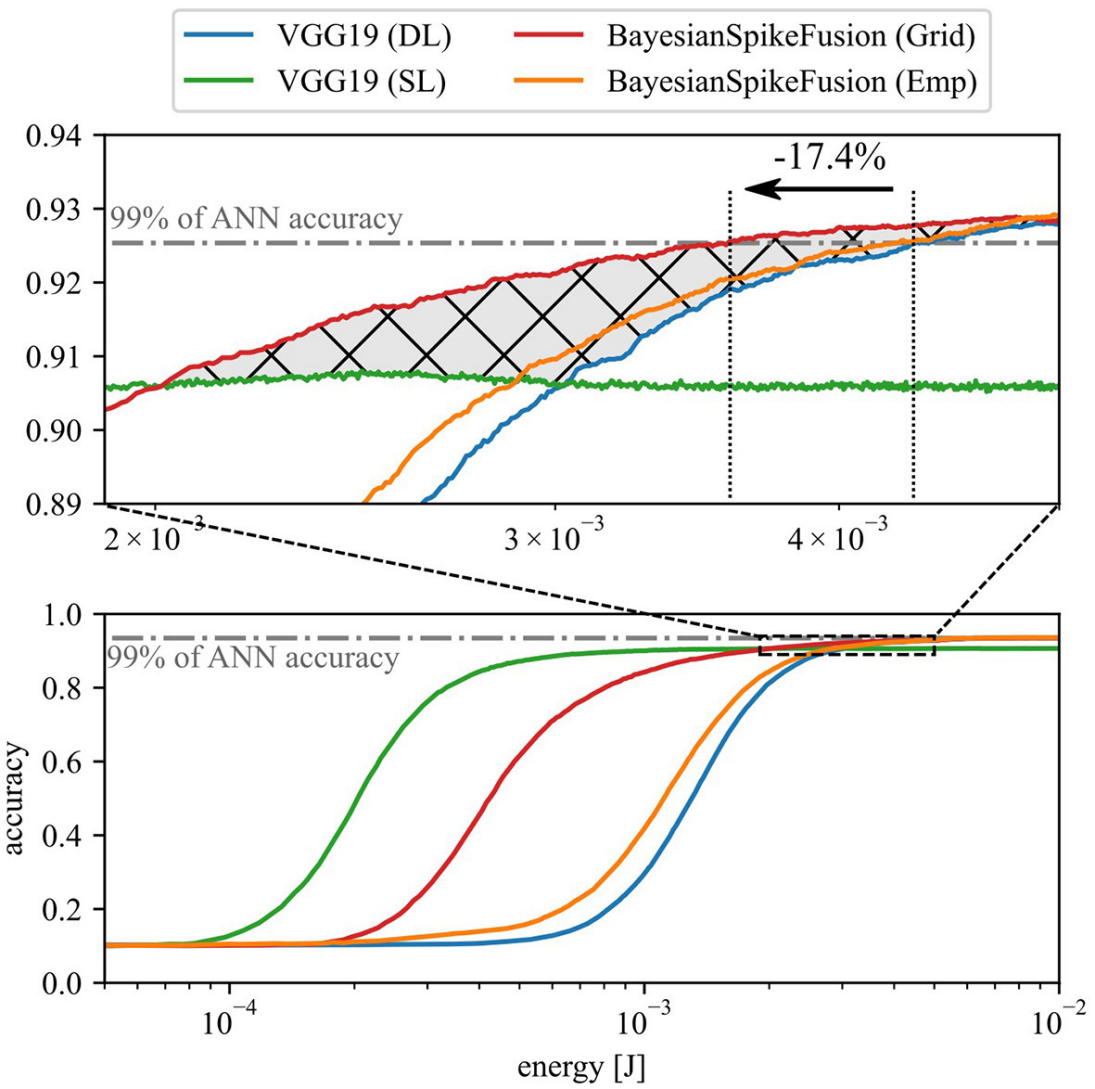
Relationship between inference accuracy and energy consumption in CIFAR10 inference using VGG19 network.

Figure 8 shows the relationship between inference accuracy and energy consumption in CIFAR10 inference using VGG19. VGG19(DL) represents the complete VGG19 model, while VGG19(SL) excludes layers after the fifth layer. The horizontal line represents 99% of the accuracy achieved by the original floating-point VGG19 model before conversion to SNN. The graph also shows two types of Bayesian spiking fusion (BayesianSpikeFusion): one with α determined through empirical Bayesian estimation labeled as BayesianSpikeFusion(Emp) and the other with α determined via grid search labeled as BayesianSpikeFusion(Grid). Note that the energy consumed by BayesianSpikeFusion is the sum of the energy consumed by VGG19(DL) and the energy consumed by IC. The energy consumption of the IC is significantly lower than the total energy consumption of the network, amounting to 0.49 nJ per time step, which is less than 0.5% of the total energy consumed per timestep. According to Equation (15), the conversion error to SNN decreases inversely with the simulation time *T*. Thus, increasing *T* enhances inference accuracy but also raises the number of spike firings, resulting in higher energy consumption. Moreover, in terms of the rise in accuracy with respect to energy consumption, VGG19(DL) demonstrates the slowest increase, while VGG19(SL) exhibits the fastest rise. Conversely, when sufficient energy is expended, VGG19(DL), BayesianSpikeFusion(Grid), and BayesianSpikeFusion(Emp) asymptotically approach the same inference accuracy as the ANN counterpart, while VGG19(SL) achieves less than 91% accuracy. This implies that, for VGG19(DL), spikes should travel through tens of layers to be accumulated at the membrane of the output neurons, and hence it takes more time to produce accurate outputs, resulting in a slower increase in inference accuracy. On the other hand, VGG19(SL) enables early propagation of information, leading to a quicker rise in inference accuracy owing to fewer number of layers. However, due to its shallow depth, VGG19(SL) lacks the discriminative ability necessary to achieve sufficient inference accuracy even with increased energy consumption.

To address this issue, BayesianSpikeFusion aims to achieve both high inference performance equivalent to ANN and a steep rise in inference accuracy by integrating VGG19(DL) and VGG19(SL) using Bayesian fusion. The graph illustrates that both BayesianSpikeFusion(Grid) and BayesianSpikeFusion(Emp) achieve high inference accuracy with lower energy consumption than VGG19(DL), effectively improving the trade-off between inference accuracy and energy consumption. Specifically, to achieve 99% of the inference accuracy of ANN counterpart, VGG19(DL) required 4.33 mJ while BayesianSpikeFusion(Grid) required only 3.58 mJ, leading to a 17.4% reduction.

To quantitatively compare the trade-off between inference accuracy and energy consumption, the area under the curve (AUC) was calculated for the curve enclosed by the curve and the lines *y* = 0 and *x* = 1.50 × 10^−2^. A larger AUC indicates that high accuracy can be achieved with less energy consumption. BayesianSpikeFusion achieved an AUC of 1.351 × 10^−2^, representing a 1.01 times improvement compared to VGG19(SL)'s AUC of 1.340 × 10^−2^, and a 1.05 times improvement compared to VGG19(DL)'s AUC of 1.281 × 10^−2^. This indicates an enhancement in the trade-off between inference accuracy and energy consumption.

In conventional early-exit methods, switching between IC and FC was based solely on confidence level. That is when classifying based on FC, information from IC was discarded. Therefore, the energy consumption curve when adopting the early-exit approach corresponds to the higher of either the energy-accuracy curve for IC or FC (represented by the green IC curve and blue FC curve connected at the intersection in Figure 8). In contrast, the red curve of BayesianSpikeFusion (Grid) surpasses this curve in terms of accuracy in the range from *x* = 2.0 × 10^−3^ to *x* = 5.0 × 10^−3^, increasing the AUC by 1.66 × 10^−4^ (corresponding to the cross-hatched area in the Figure 8). Therefore, BayesianSpikeFusion demonstrates higher energy efficiency compared to early-exit methods that utilize results from only one IC or FC for classification.

**Figure 9:**
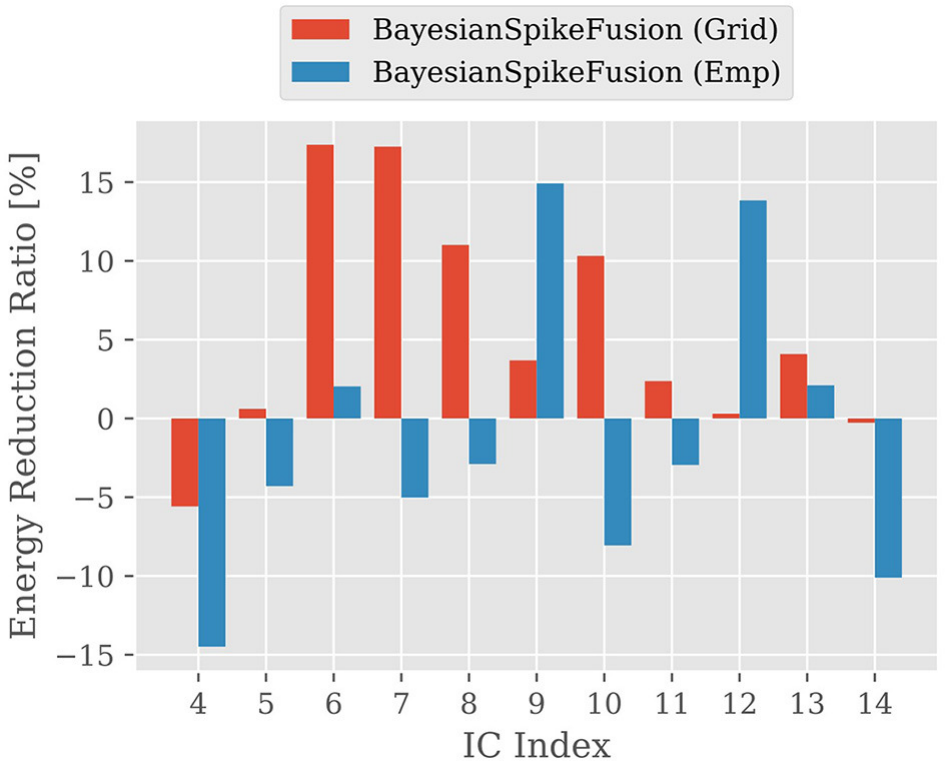
The reduction ratio of energy consumption per image; The vertical axis shows the reduction ratio and the horizontal axis shows the IC insertion position, comparing the reduction ratio of energy consumption required for the SNN to reach 99% of the accuracy of the ANN before conversion.

Next, we investigated the impact of the insertion position of the IC on inference accuracy and energy consumption. Figure 9 illustrates the reduction rate (*R*) of energy consumption for inference, while shifting the insertion position of the IC from the layer after the fourth convolutional layer to the layer after the fourteenth convolutional layer, one layer at a time. *R* is defined as follows Equation (33):


(33) $$\begin{array}{ll} R & =100\cdot \frac{E_{\text{orig}}-E_{\text{prop}}}{E_{\text{orig}}}, \end{array}$$

where *E*_orig_ and *E*_prop_ represent the energy required for the VGG19 converted to SNN and the VGG19 using BayesianSpikeFusion, respectively, to achieve 99% of the inference accuracy of the original ANN before conversion to SNN.

From Figure 9, it can be observed that inserting the IC at both shallow and deep positions does not yield sufficient effectiveness. This is because when the IC is inserted in shallow layers, there are insufficient features acquired in those layers to predict the firing probability of the output layer, resulting in a deterioration in the construction accuracy of the prior distribution and hindering the improvement of accuracy through Bayesian fusion. On the other hand, even when the IC is inserted in deep layers, it takes time for information to propagate sufficiently to the neurons in the deep layers, thereby unable to take full advantage of the benefits of Bayesian fusion. Therefore, it is evident that inserting the IC at the appropriate position is crucial.

We compared the energy consumption and AUC of an SNN and BayesianSpikeFusion that were pre-charged with an initial membrane potential of 0.5. The results of the inference of CIFAR-10 with the VGG19 model are shown on the top side of Table 3. From these results, in our experimental settings, setting the initial membrane potential to 0.5 resulted in a deterioration of energy consumption and AUC for both the conventional SNN and BayesianSpikeFusion. We believe this is because spikes that would normally be difficult to fire are excessively fired, resulting in a deterioration of the initial inference accuracy.

**Table 3:** Inference accuracy of CIFAR-10 using VGG19 with varying initial membrane potential *V*^0^ or threshold *V*_*th*_.

	**AUC** (× 10^−3^)	**Energy** (× 10^−3^) **[J]**
*V*^0^ **Experiment**		
Conventional SNN	2.84	4.33
Conventional SNN (*V*^0^ = 0.5)	2.60 (↓−8.5%)	5.06 (↑−16.9%)
BayesianSpikeFusion	3.53	3.58
BayesianSpikeFusion (*V*^0^ = 0.5)	3.47 (↓−1.8%)	4.59 (↑−28.2%)
*V*_*th*_ **Experiment**		
Conventional SNN	2.84	4.33
Conventional SNN (*V*_*th*_ = 0.8)	2.72 (↓−4.2%)	4.59 (↑−5.9%)
Conventional SNN (*V*_*th*_ = 1.2)	2.91 (↑2.7%)	4.11 (↓5.0%)
BayesianSpikeFusion	3.53	3.58
BayesianSpikeFusion (*V*_*th*_ = 0.8)	3.52 (↓−0.4%)	4.10 (↑−14.7%)
BayesianSpikeFusion (*V*_*th*_ = 1.2)	3.51 (↓−0.7%)	3.55 (↓0.8%)

In addition, we compared the energy consumption and AUC of an SNN and BayesianSpikeFusion with thresholds set to 0.8 and 1.2. The results of the inference of CIFAR-10 with the VGG19 model, similar to the previous experiment, are shown on the bottom side of Table 3. From Table 3, an SNN with a threshold set to 0.8 resulted in a decrease in AUC and an increase in energy consumption for both the conventional SNN and BayesianSpikeFusion. We believe this is due to the excessive firing of spikes, similar to the case where the initial membrane potential was pre-charged. On the other hand, an SNN with a threshold set to 1.2, although not as good as BayesianSpikeFusion alone, increased the AUC by 2.7% and reduced energy consumption by 5.0% compared to the conventional SNN. BayesianSpikeFusion with a threshold set to 1.2 had the same AUC and energy consumption as the regular BayesianSpikeFusion with a threshold of 1.0, and no synergistic effect was observed.

Finally, to investigate the generality of BayesianSpikeFusion, we examined the required inference energy to achieve 99% of the inference accuracy of the ANN counterpart for five network architectures: VGG11, VGG16, VGG19, ResNet18, and ResNet34, using three datasets: MNIST, CIFAR10, and CIFAR100. The results are shown in Table 4. Additionally, AUC was calculated to quantify the trade-off between inference accuracy and energy consumption, and the values were added to the table. From Table 4, it can be seen that BayesianSpikeFusion(Grid) achieved energy reduction in all conditions. On the other hand, BayesianSpikeFusion(Emp) showed increased energy consumption in some conditions (CIFAR-10+ResNet18, CIFAR-100+VGG11, and CIFAR-100+ResNet18), but achieved equal or higher AUC values. In addition, we investigated the AUC and energy when inferring Tiny ImageNet (Le and Yang, 2015), which is a larger dataset than the three datasets, with the VGG19 model, and the results are shown in Table 5. Tiny ImageNet is composed of a part of the ImageNet data and is a set of 100,000 images of 200 classes (500 images each) reduced to 64 × 64 color images. From Table 5, both search methods (i.e., empirical Bayes method and grid search) improved the trade-off between energy and accuracy and were able to reduce energy consumption. We also evaluated the DVS Gesture dataset (Amir et al., 2017), which consists of time-series neuromorphic data. This dataset includes 11 types of hand gestures performed by 29 subjects in three different environments. In experiments using VGG19, BayesianSpikeFusion (Grid) reduced energy consumption by 50.0% compared to conventional SNNs, while BayesianSpikeFusion (Emp) reduced energy consumption by 43.7%. This demonstrates that the proposed method is also effective for neuromorphic datasets with time-series information.

**Table 4:** Comparison of AUC and energy consumption.

**Architecture**	**Method**	**Accuracy [%]**	**AUC** **(× 10^**−3**^)**		**Energy** **(× 10^**−3**^)** **[J]**	
**MNIST**				
VGG11	Conventional SNN	**98.58**	0.11		0.24	
	BayesianSpikeFusion(Grid)	98.57	**0.12**	**(↑8.2%)**	**0.23**	**(↓3.4%)**
	BayesianSpikeFusion(Emp)	**98.58**	0.11	(↑ 1.2%)	0.24	(↓ 0.3%)
VGG16	Conventional SNN	98.65	0.48		0.98	
	BayesianSpikeFusion(Grid)	98.64	**0.71**	**(↑49.8%)**	**0.76**	**(↓22.7%)**
	BayesianSpikeFusion(Emp)	**98.67**	0.54	(↑ 13.1%)	0.89	(↓ 8.5%)
VGG19	Conventional SNN	98.59	0.84		1.64	
	BayesianSpikeFusion(Grid)	**98.62**	**1.24**	**(↑47.7%)**	**1.00**	**(↓38.8%)**
	BayesianSpikeFusion(Emp)	98.59	0.89	(↑ 5.6%)	1.55	(↓ 5.1%)
ResNet18	Conventional SNN	**98.69**	1.08		2.36	
	BayesianSpikeFusion(Grid)	98.66	**1.80**	**(↑66.6%)**	**1.70**	**(↓27.8%)**
	BayesianSpikeFusion(Emp)	98.67	1.26	(↑ 16.2%)	2.18	(↓ 7.5%)
ResNet34	Conventional SNN	**98.65**	10.49		21.66	
	BayesianSpikeFusion(Grid)	**98.65**	**16.39**	**(↑56.2%)**	**16.65**	**(↓23.1%)**
	BayesianSpikeFusion(Emp)	**98.65**	10.93	(↑ 4.2%)	19.95	(↓ 7.9%)
**CIFAR-10**				
VGG11	Conventional SNN	90.74	0.65		0.93	
	BayesianSpikeFusion(Grid)	90.73	**0.70**	**(↑8.0%)**	**0.86**	**(↓7.7%)**
	BayesianSpikeFusion(Emp)	**90.75**	0.67	(↑ 3.0%)	0.90	(↓ 3.5%)
VGG16	Conventional SNN	**92.81**	1.89		2.76	
	BayesianSpikeFusion(Grid)	92.79	**2.20**	**(↑16.5%)**	**2.28**	**(↓17.4%)**
	BayesianSpikeFusion(Emp)	92.80	1.96	(↑ 4.0%)	2.34	(↓ 15.0%)
VGG19	Conventional SNN	**92.55**	2.84		4.33	
	BayesianSpikeFusion(Grid)	**92.55**	**3.53**	**(↑24.6%)**	**3.58**	**(↓17.4%)**
	BayesianSpikeFusion(Emp)	92.54	2.98	(↑ 5.1%)	4.24	(↓ 2.0%)
ResNet18	Conventional SNN	**93.53**	6.72		9.51	
	BayesianSpikeFusion(Grid)	**93.53**	6.88	(↑ 2.4%)	**9.38**	**(↓1.4%)**
	BayesianSpikeFusion(Emp)	**93.53**	**6.89**	**(↑2.6%)**	9.76	(↑ -2.6%)
ResNet34	Conventional SNN	93.87	21.30		35.14	
	BayesianSpikeFusion(Grid)	**93.90**	**28.30**	**(↑32.9%)**	**30.30**	**(↓13.8%)**
	BayesianSpikeFusion(Emp)	93.88	22.14	(↑ 3.9%)	34.26	(↓ 2.5%)
**CIFAR-100**				
VGG11	Conventional SNN	**68.84**	0.97		1.74	
	BayesianSpikeFusion(Grid)	**68.84**	**0.99**	**(↑2.2%)**	**1.59**	**(↓9.1%)**
	BayesianSpikeFusion(Emp)	**68.84**	0.98	(↑ 0.7%)	1.85	(↑ -5.9%)
VGG16	Conventional SNN	70.88	2.57		4.77	
	BayesianSpikeFusion(Grid)	**70.89**	**2.88**	**(↑12.1%)**	**4.48**	**(↓6.1%)**
	BayesianSpikeFusion(Emp)	**70.89**	2.61	(↑ 1.6%)	4.53	(↓ 5.1%)
VGG19	Conventional SNN	71.06	4.39		8.48	
	BayesianSpikeFusion(Grid)	71.07	**4.99**	**(↑13.5%)**	7.51	(↓ 11.4%)
	BayesianSpikeFusion(Emp)	**71.11**	4.44	(↑ 1.1%)	**7.12**	**(↓16.0%)**
ResNet18	Conventional SNN	**73.73**	6.04		10.99	
	BayesianSpikeFusion(Grid)	73.72	**6.39**	**(↑5.8%)**	**10.85**	**(↓1.3%)**
	BayesianSpikeFusion(Emp)	**73.73**	6.05	(↑ 0.2%)	11.15	(↑ -1.5%)
ResNet34	Conventional SNN	71.01	50.19		88.73	
	BayesianSpikeFusion(Grid)	71.01	**53.94**	**(↑7.5%)**	**46.11**	**(↓48.0%)**
	BayesianSpikeFusion(Emp)	**71.02**	50.52	(↑ 0.7%)	66.89	(↓ 24.6%)

The values written in bold indicate the best performance (i.e., accuracy, AUC, or energy consumption) when comparing each method under the same dataset and model conditions.

**Table 5:** Inference accuracy of Tiny ImageNet using VGG19.

	**VGG19**	**Bayesian SpikeFusion (Grid)**	**Bayesian SpikeFusion (Emp)**
Accuracy [%]	64.9	65.0	64.9
AUC (× 10^−3^)	21.70	**23.14** (↑6.6%)	21.82 (↑0.6%)
Energy (× 10^−3^) [J]	41.49	**38.89** (↓6.3%)	41.12 (↓0.9%)

The values written in bold indicate the best performance (i.e., accuracy, AUC, or energy consumption) when comparing each method under the same dataset and model conditions.

Table 6 compares BayesianSpikeFusion with other methods for reducing inference time steps on the CIFAR-10 dataset using VGG16. It shows that BayesianSpikeFusion can achieve similar accuracy while reducing time steps and energy consumption compared to Robust normalization and RMP. Compared to RTS, BayesianSpikeFusion also achieves comparable accuracy. Furthermore, it is noted that BayesianSpikeFusion can be used concurrently with these methods, potentially further reducing the inference time steps.

**Table 6:** Performance comparison of CIFAR-10 dataset inferred by VGG16.

**Method**	**Accuracy [%]**	**Energy [J]**	**Timestep**
Robust normalization (Rueckauer et al., 2017)	93.77	1.99 × 10^−2^	2500
RMP (Han et al., 2020)	93.63	1.22 × 10^−2^	1536
RTS (Deng and Gu, 2021)	92.24	1.02 × 10^−3^	128
BayesianSpikeFusion(Grid)	93.57	8.14 × 10^−3^	1024
BayesianSpikeFusion(Grid)	92.22	2.05 × 10^−3^	256
BayesianSpikeFusion(Grid)	88.14	1.02 × 10^−3^	128

## 7 Conclusion

We proposed a method for reducing the energy required for SNN inference by predicting the firing probability of the final layer from activations in the shallow layers of the network and Bayesian fusion of these activations with actually observed firing events.

To evaluate the effectiveness of the proposed method, we implemented VGGs and ResNets with PyTorch and trained image classification tasks on MNIST, CIFAR10, and CIFAR100. Comparing the energy required to achieve a 1% degradation in classification accuracy from the ANN's inference accuracy, we achieved a maximum reduction of 42.62mJ and a 48.0% energy reduction in ratio.

## Data availability statement

Publicly available datasets were analyzed in this study. This data can be found here: MNIST (http://yann.lecun.com/exdb/mnist/), CIFAR-10/CIFAR-100 (https://www.cs.toronto.edu/~kriz/cifar.html), Tiny ImageNet (https://www.kaggle.com/c/tiny-imagenet) and the DVS Gesture dataset (https://ibm.ent.box.com/s/3hiq58ww1pbbjrinh367ykfdf60xsfm8/folder/50167556794).

## Author contributions

TH: Writing – review & editing, Visualization, Validation, Software, Methodology, Investigation, Formal analysis, Data curation, Conceptualization. TS: Writing – review & editing, Supervision, Resources. HA: Writing – original draft, Resources, Project administration, Funding acquisition.
